# MolabIS - An integrated information system for storing and managing molecular genetics data

**DOI:** 10.1186/1471-2105-12-425

**Published:** 2011-10-31

**Authors:** Cong VC Truong, Linn F Groeneveld, Burkhard Morgenstern, Eildert Groeneveld

**Affiliations:** 1 Department of Breeding and Genetic Resources, Institute of Farm Animal Genetics (FLI), Neustadt, Germany; 2 Department of Primatology, Max Planck Institute for Evolutionary Anthropology, Leipzig, Germany; 3 Department of Bioinformatics, Institute of Microbiology and Genetics, University of Göttingen, Germany

## Abstract

**Background:**

Long-term sample storage, tracing of data flow and data export for subsequent analyses are of great importance in genetics studies. Therefore, molecular labs do need a proper information system to handle an increasing amount of data from different projects.

**Results:**

We have developed a molecular labs information management system (MolabIS). It was implemented as a web-based system allowing the users to capture original data at each step of their workflow. MolabIS provides essential functionality for managing information on individuals, tracking samples and storage locations, capturing raw files, importing final data from external files, searching results, accessing and modifying data. Further important features are options to generate ready-to-print reports and convert sequence and microsatellite data into various data formats, which can be used as input files in subsequent analyses. Moreover, MolabIS also provides a tool for data migration.

**Conclusions:**

MolabIS is designed for small-to-medium sized labs conducting Sanger sequencing and microsatellite genotyping to store and efficiently handle a relative large amount of data. MolabIS not only helps to avoid time consuming tasks but also ensures the availability of data for further analyses. The software is packaged as a virtual appliance which can run on different platforms (e.g. Linux, Windows). MolabIS can be distributed to a wide range of molecular genetics labs since it was developed according to a general data model. Released under GPL, MolabIS is freely available at http://www.molabis.org.

## Background

Recent advances in molecular genetics have led to a widespread use of molecular markers in genetic research for both animals and plants [1-3]. Particularly, microsatellite genotyping [4-6] and Sanger sequencing [7-9] are being widely used for different objectives in small-to-medium sized labs for biodiversity studies. DNA sequencing and microsatellite genotyping experiments often go through several major steps such as sample collection, DNA extraction, PCR amplification, electrophoresis and result analysis. Fundamental principles for conducting experiments are given in textbooks or technical documentation. Normally, lab users develop their own procedures, which they describe in lab protocols, to carry out lab work at each step. In other words, protocols provide essential information, such as how to prepare samples, what materials are needed, how to setup the machine, and what information to collect for workflow support, etc. for the completion of lab work. Although different labs may perform similar steps, the data processing operations at each step are not necessarily the same. Moreover, the demand for storage, use and management of data varies lab by lab. Therefore, identifying data items for data storage is essential. For the development of integrated information systems applicable to a wide range of labs, a general data model must be designed in the first phase. This data model must meet all requirements of different labs without additional programming or modification. In the second phase, the required functionality must be implemented resulting in a general software package.

We have previously developed a formalized workflow [10] and a data framework to concretely describe pipelined data processes and data items generated at each step which serves as the basis for the database design in the first phase. Accordingly, in these contributions, the term "workflow" specifies the flow of operations (or tasks) relevant to data, not actual lab work steps. In other words, we only focus on the workflow for capturing and handling data. At each step of the workflow, we use a "data integration table" (DIT) to represent data items required in labs. Each DIT is a table with n rows and m columns where the values in the columns of each row specify names, data types, data sources and requirements of surveyed labs, respectively. The collection of these DITs forms a data framework which helps us to construct the general data model for developing MolabIS. The details, which focus on the construction of DITs as well as the methodology for building the formalized data framework, will be presented in another contribution.

### Data handling in molecular biology labs

The challenges that small-to-medium sized labs face can be classified into five major issues. First, searching and keeping track of data is often inefficient, since heterogeneous data, possibly from different sequencers, is stored and managed in a non-standard way. Each scientist has her or his own way to handle data. Often, there is no naming convention among scientists for data objects such as individuals or samples. Second, it is difficult to share and merge data generated by different persons, because data is isolated among scientists and projects. In practice, data is often scattered and stored in inconvenient formats. Some information may be stored in paper lab books, whereas other data are kept in file systems. Third, due to the lack of a centralized database, making reports becomes difficult for project managers, because too much time has to be spent on combining data sets from various sources and locations. Fourth, sometimes data cannot be found and is thus lost. This problem is most prominent in labs with short term lab users like master or doctoral students. Typically, they come to the lab with their samples and leave the lab with their data. Fifth, scientists often spend much time on manually preparing and converting data. In order to start lab work such as PCR amplification or electrophoresis, a scientist has to know the availability and physical location of samples. This information is often found in a paper lab book, which may be difficult to retrieve. In addition, conversion and compilation of data for further analyses is carried out manually, which is, both time consuming and prone to error. Most of these challenges are often prominent in labs conducting biodiversity experiments, since sharing and synthesis of data among projects are regular incidents.

### Requirements

To address the above challenges, we developed a proper information system for long-term data storage. It comprises essential tools to handle, retrieve, report and convert data effectively with a focus on biodiversity experiments. Such an information system must meet specific requirements as follows:

**R1: **The information system stores and manages sequence and microsatellite data of different projects in small-to-medium sized labs conducting Sanger sequencing and microsatellite genotyping experiments.

**R2: **It supports the management of individuals from which samples were derived, including their classification into species and breeds or varieties.

**R3: **Sample management is provided to keep track of all kinds of material (e.g. blood, tissue) from different projects collected by different users. The sample storage scheme is suitable for any physical storage location of samples in different labs.

**R4: **The information system provides functionality for managing the workflow and the traceability of samples in lab procedures. It allows tracing lab work such as DNA extraction, polymerase chain reaction (PCR), PCR validation, and electrophoresis to capture all original data from possibly different machines.

**R5: **The information system supports basic functionality (searching, viewing, retrieving and modifying) and the import of large amounts of samples, sequences and microsatellites from external files. Raw data received from different architectures of sequencers can be stored and retrieved in a uniform way.

**R6: **Ready-to-print reports can be generated easily to provide data and statistics of a certain project or an entire database.

**R7: **Sequences and microsatellites (final data) can be converted to various data formats for further analyses.

**R8: **The information system is a multi-user system which supports security and access control.

**R9: **The software package runs on different platforms (e.g. Linux, Windows) with a simple installation procedure which allows users with no experience in programming and database management to setup and use the system. The software is freely available to be used, distributed, and modified without restrictions. Therefore, open-source software, e.g. under the GPL license, is preferred.

**R10: **Migration of data from previous projects is supported by the software package.

### Existing information systems

In recent years, biologists, bioinformaticians and computer scientists have spent much effort to confront the challenges of storing and managing heterogeneous data in a uniform way [11]. Therefore, a whole class of software systems has been developed to support lab work, appropriately called Lab Information Management Systems or LIMS. It has to be noted that there are many types of labs with different requirements for data storage and management. Accordingly, LIMS developed for a chemistry lab will support very different work than a LIMS required in a molecular genetic lab. In the latter class, a number of LIMS developments have been reported. Most of them focused on the storage and management of processed data including microarray [12-14] and proteomics data [15-17]. Wendl et al. [18] developed an information system to keep track of sequencing workflows, but it does not support collecting information on individuals and microsatellite data. In 2006, a group of researchers developed AGL-LIMS [19], an open source information system for genotyping workflows which meets some of our requirements. As it focuses on microsatellite data in plants, sequencing is not supported. Further, the management of individuals, original samples along with the physical storage places are not considered. Recently, some database applications were devoted to the management of both Single Nucleotide Polymorphisms (SNP) genotype data and phenotype data [20,21]. Additionally, Weiβensteiner et al. extended their system developed in 2009 [21] to enable the import and storage of mtDNA and STR (Short Tandem Repeats) data [22]. In 2010, Ducan et al. also provided an open source web application to enable researchers to store, organize and retrieve their sequence data [23].

In general, the common objective of these information systems is to provide means for lab users to keep their data in-house and extract data for further analyses. However, they often aim to capture raw data from a specific platform [20], or import only final data, while ignoring raw data [22,23]. Most of them do not support the management of individuals and traceability of samples in lab procedures. Some systems [21-23] do not provide a solution for documenting lab data.

Since available information systems are designed in a specific context of a lab, installation and use in other labs is usually a challenge. To the best of our knowledge, there is no LIMS available, which meets all requirements stated above. We have therefore designed a general data model for labs conducting Sanger sequencing and microsatellite genotyping. In this paper, we present the design, implementation and features of MolabIS, an integrated information system for storing and managing sequence and microsatellite data in molecular genetics labs with a focus on biodiversity experiments.

## Implementation

### Database design

**Figure 1:**
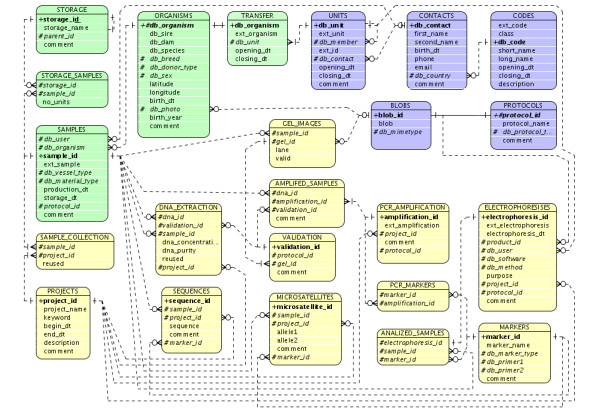
**Entity-Relationship diagram of MolabIS**. Entity-Relationship diagram using Crow's Foot notation presents the conceptual data structure used in MolabIS. Entities and relationships are represented as boxes and lines between the boxes, respectively. The database structure consists of 23 tables presented in three groups (three different colors). To simplify the complexity of the data model, foreign keys which are linked to Codes and Protocols are not shown.

The first step in database design is the definition of a data model. In order to build such a data model, we need to know (1) differences in data streams in labs, (2) data types spawned from those data streams, (3) what data items should be stored at each step, and (4) how lab users use and retrieve their data. Figure 1 shows the conceptual database structure of MolabIS in form of the Entity-Relationship diagram (ERD) [24] using Crow's Foot notation. Specifically, the database structure could be divided into three groups of closely linked relations (tables). The first group consists of five tables (**codes**, **unit**, **contacts**, **blobs**, and **protocols**) which are used to store initial data, information on lab users and experimental protocols. The **codes **table keeps the references of foreign keys in the information system. Instead of using many tables to store foreign keys of different types, we grouped them together in one table. A column called "class" in the table **codes **stores classes of foreign keys such as SPECIES, or BREED. Table 1 lists 14 such classes used in MolabIS. Typically, each class is a drop-down list in the data entry forms of MolabIS. Each value in the class (a code) is a data item from the drop-down list. Therefore, whenever a user wants to choose a data item, which is not available in such drop-down lists (e.g. species), he or she should insert a new code for the corresponding class. Two tables **unit **and **contacts **manage all contacts stored in the database. By storing the content of files as binary large objects (BLOBS), all lab protocols are managed in the database via two tables **protocols **an **blobs**.

**Table 1:** Classes in the codes table

#	Class	Description
1.	BREED	breeds of animals or varieties of plants
2.	COUNTRY	countries of users or contacts
3.	LANGUAGE	speaking languages of users or contacts
4.	MARKER_TYPE	types of molecular markers
5.	MATERIAL_TYPE	types of biological materials
6.	METHOD	electrophoresis methods for sequencing
7.	MIMETYPE	types of file extension
8.	PRIMER	names of PCR primers
9.	PROTOCOL_TYPE	types of experimental protocols
10.	PURPOSE	sequencing or genotyping
11.	SEX	genders of individuals
12.	SOFTWARE	software tools are used to analyze data
13.	SPECIES	species of individuals
14.	VESSEL_TYPE	types of vessels for storing samples

The **codes **table provides fourteen classes to keep the references of foreign keys. Each class has many different values. The values are used to make drop-down lists in the data entry forms.

**Figure 2:**
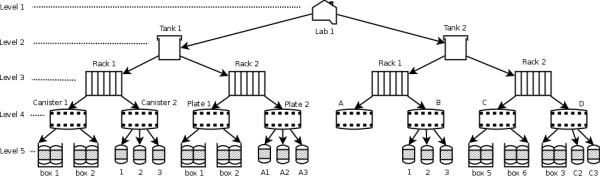
**Management of sample storage**. A five level storage schema is used to manage the location of samples. It is constructed as a tree model. The highest level (level 1) is the storage location. The lowest level (level 5) is the sample storage. The middle levels (level 2, level 3 and level 4) may be different among labs. The labels in all levels are defined by users.

The second group with five tables (**organisms**, **transfer**, **storage**, **samples**, **storage-samples**) manages data on individuals, samples and DNA. The combination of two tables **organisms **and **transfer **allows us to store the detail of all individuals of any species and breed or variety. It also helps to accept any external identification system of animals or plants. Tracking of samples is conducted with the triplet of **samples**, **storage**, and **storage-samples**. Sample storage is managed by a five level hierarchy, creating a storage tree (see Figure 2, explained in detail later), in which each location has a single parent (upper location) and many children (lower locations). In relational databases, this data structure is organized in a single table with three columns "storage-id", "storage-name", and "parent-id" as in the **storage **table.

The last group consists of several tables which, deal with tracking the workflow. The collection of samples and the extraction of DNA are managed in tables **sample-collection **and **dna-extraction**, respectively. In addition to storing information on DNA, the **dna-extraction **also saves the traces of the original samples extracted. The details of PCR amplification and electrophoresis are recorded in the tables **pcr-amplification**, **pcr-markers**, **amplified-samples **and **electrophoresis**. Two tables **validation **and **gel-images **are used to store the information on the validation of DNA or PCR products and the content of gel images. Final data is stored in the two tables **sequences **and **microsatellites**.

In order to derive a general data model, two important points have been considered. First, the data model allows for storage of different data types of original data regardless of the hardware variations of sequencers. The database was designed on an abstract level to accept any type of raw files, for instance, gel images of a gel electrophoresis, or chromatogram files of capillary electrophoresis. Instead of using many different tables to serve different data types, all raw data files are stored as BLOBs in a single table. Second, the data model only comprises elements which are at least in principle available for every species, sample type, and lab. Other more specific elements can be stored in text blocks and BLOBs. As a result, the data model can be applied without customization to capture data of any species, breed (or variety), biological material type and hierarchical sample storage scheme.

### Application architecture

**Figure 3:**
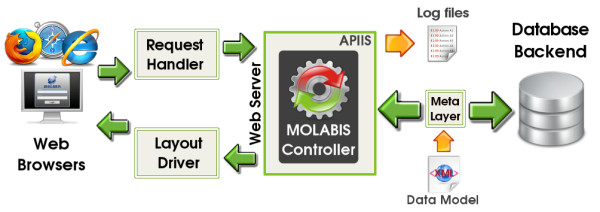
**Application architecture**. MolabIS is based on a three-tier client/server architechture including presentation tier, application tier and data tier.

MolabIS is an integrated information system which is developed on the basis of APIIS [25], a framework for developing adaptable platform independent information systems. It is a web application based on a three-tier client/server architecture (see Figure 3). On the client side, end-users from any computer in the local area network (LAN) can interact with the system to carry out all activities via a standard web browser (e.g. Firefox, Internet Explorer) on any operating system. No additional software packages or programs need to be installed on the client machine. The incorporation of Web 2.0 technologies such as Ajax [26] makes web interactions simpler and more effective. The menu bar helps end-users to easily navigate all web forms. Web layouts and dynamic interactions are controlled by Javascript, CSS (Cascading Style Sheets) and Prototype (an Open Source Javascript Framework) [27] to create an easy-to-use graphical user interface (GUI).

At the data tier, Postgres [28], an open source database management system (DBMS), is used to store application data and handle all data transactions. The application tier requires an Apache web server [29] running under the Linux operating system. On the top of APIIS [25], the MolabIS controller is central to the application tier to process user requests and to communicate with other components. The application source code is written mainly in the Perl programming language [30]. Many Perl modules, which are available on CPAN [31], are used to implement different functionalities in the system. The APIIS meta layer between the web server and the database server controls data transactions and error handling. Many open source software packages are integrated in MolabIS. Particularly, HTML::Templates [32] and CGI::Ajax [33] are two Perl modules used to produce and handle dynamic web forms. Since our objective is to have a uniform layout, form templates are all designed in the same manner. They are compiled by the MolabIS controller to create web pages, which are sent to the web browsers. The labels of form elements in each form template are variables translated from a text file in ASCII format, allowing easy changes of labels on the forms. The forms are designed so that a large number of data records (e.g. samples, DNA) can be entered, imported and processed. Because of its dynamic length, the form has to be broken down into smaller units called sub forms. A data buffer is implemented on the server to ensure the temporary storage of data of sub forms before they are submitted to the database.

As an APIIS application, the database of MolabIS is created from a XML (eXtensible Markup Language) [34] schema called "model file". The model file also defines a set of business rules for each table in the database. These business rules are checked at the meta layer in the APIIS framework to guarantee atomicity and consistency [35].

We selected an automatic report generation solution in JasperReports [36], an open source reporting library written in Java, to make ready-to-print reports in PDF format. It is integrated into the MolabIS controller with the assistance of the Inline::Java package [37]. JasperReports templates in XML were designed under iReport [38], an intuitive and visual report editor for JasperReports. These templates can be customized and checked independently without affecting the application code. Further, BioPerl [39] was used to support converting sequence data to a number of specific formats.

### Security

The information system must provide mechanisms for user authentication to protect data from unauthorized accesses, according to the design requirements. Since users may play different roles in the system, they should accordingly be granted different rights for the utilization of the system and its data. The system controls the access of a user to functionality and data once he or she logged in successfully through "user roles". Each role is a definition of a group of access rights to determine which part of the program is hidden or shown. They also define which part of the database can be accessed and modified by the end-user. In our application, user roles are considered on both levels of system and database to assign proper tasks. Therefore, after a user account is created it has to be granted one "user role on the system tasks" (SR) and one "user role on the database tasks" (DR).

There are four SRs corresponding to four kinds of users. Each SR in this case is assigned a given number of system tasks depicted in Table 2. While the management of SRs handles access rights for different functions or modules of the application, the management of DRs is responsible for checking all activities related to the content of the database. Table 3 lists five DRs along with expected data access rights.

**Table 2:** User rights on system functionality

	User role	(a)	(b)	(c)	(d)	(e)	(f)	(g)
1.	User administrator	•						•
2.	Lab manager			•	•	•	•	•
3.	Scientist		•	•	•	•		•
4.	Visitor			•	•	•		•

Each row defines access rights to seven functional blocks (a: manage users, b: use workflow, c: update data, d: generate reports, e: export data, f: administrate data, g: get help). *User administrators *can add users to or remove users from the system. They can update the data and grant new roles to existing users. *Lab managers *can use most of the functions in the system except data entry via the workflow. *Scientists *can deal with the workflow for data entry and use other functions of the system except the administration of common data in the lab. *Visitors *can use some functions such as viewing data, generating reports, converting data and reading helps. However, they are limited to work with the workflow and the administration.

**Table 3:** User rights on database manipulation

#	User role	Rights
1.	Read	access to application data
2.	Write	read and update application data
3.	Delete	remove application data
4.	Manage User	access and modify data related to users
5.	Full right	all of the above rights

Defining user roles on database tasks.

### Sample tracking and management

Often sampling individuals (animals or plants) is the first phase of molecular genetics projects. Here we use the term "sample" to imply biological material, such as blood, semen, oocytes, embryos, somatic cells, or tissue from which DNA is extracted. Sample management allows recording three blocks of information: origin of sample, sample information, and the storage location of the sample.

The first block records data of individuals from which the samples are collected. Here, samples from any species and breed (or variety in plants) are accepted. The second block specifies the sample itself. A sample is collected from a certain type of biological material on a given date by a given person. Different types of biological material result in different types of vessels and different storage units (e.g. volumes of fresh blood in vial, units of dried blood on filter paper or weight of tissue sample in a tube). The final block describes when and where the samples are stored.

Sample storage is based on the storage facility and infrastructure of each lab. Therefore, our storage management system is designed to handle physical storage in a general way by providing a five level hierarchy. This flexible storage scheme is also used to manage the location of samples in national genebanks [40] and is also used for storing DNA in MolabIS. Normally, the highest level (level 1) is used for the storage location (e.g. labs, rooms). The lower levels could define various storage facilities (e.g. tanks, shelves, racks, canisters, etc.), while the lowest is the sample storage level in which the samples can be located by sequential search. Figure 2 is an example for defining the sample storage in a small lab, where all sample containers are kept in one place. It is a storage tree where each node at each level can have multiple sub-nodes in the lower level. Each leaf node is associated to either a box of vessels or a single vessel. In such labs, we may need only four storage levels (2 to 5) to keep track of samples since there is only one node as the root of the tree in the first level. This scenario can be extended easily for large labs where samples are physically stored in different places.

Since relational databases are not well suitable to store hierarchical data, we used a tree structure to model the storage of samples in a single table (see the **storage **table in Figure 1). Technically, this helps us to take advantages of tree search algorithms for easily implementing the functionality of sample retrieval such as searching a certain sample, listing samples in a level, printing a single path of storage places.

### Data migration

One of the challenges for setting up a new information system lies in transferring large amounts of historical data collected and stored over the years to the database, prior to loading the new data into the database. Data migration is the process of transferring data from external data sources to a new database. This work can be done in either a visual loading mode or a batch loading mode. In the visual loading mode the user can employ a graphical interface to browse data from file systems, select proper data, enter related details and load everything to the database. This mode is provided in most of the information systems, and here MolabIS is not an exception, allowing this process to be carried out under the workflow. However, for large sets of data, this is time consuming, because data entry must be done manually step by step. In this case, the batch mode is more efficient. Instead of having many separated loads done manually in the visual loading mode, a big load can automatically be executed in the batch loading mode. This feature sets MolabIS apart from other information systems.

## Results

**Figure 4:**
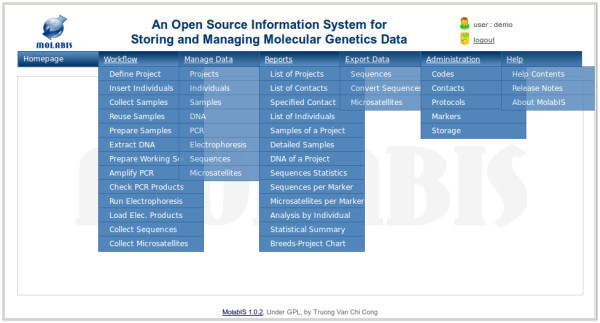
**Main user interface**. MolabIS provides five different modules which can be accessed from the menu bar in the main interface. From left to right, these modules are *Workflow*, *Manage Data*, *Reports*, *Export Data *and *Administration*. Each module consists of many sub-modules which allow users to communicate with the system via web forms.

MolabIS has been implemented as a web database application, written in approximately 40,000 lines of source code (Additional File 1). The main graphical user interface provides five different modules (Figure 4) which can be accessed from the navigation menu. All functionality has been developed to meet the requirements listed above. It provides essential tools for collecting data effiectively, searching and retrieving results easily, and making reports and extracting data quickly. The following list demonstrates the major features of MolabIS.

### Data capture and storage

**Figure 5:**
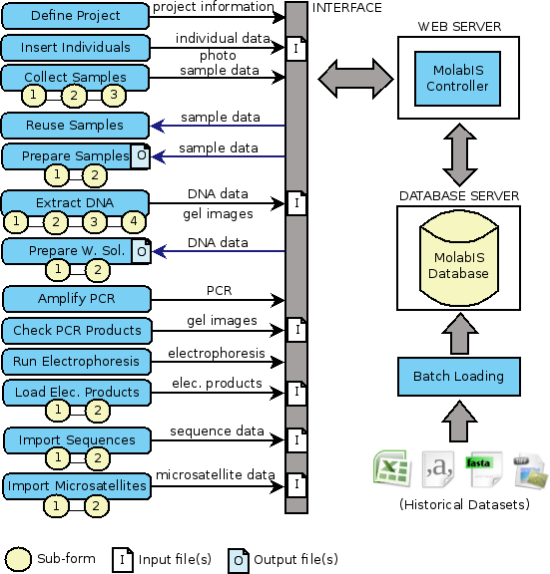
**Data flows for capturing data in MolabIS**. Data can be entered into the database through either a series of web forms under the workflow or a batch loading mode. The former is suited for inserting data of new projects while the latter is often used for data migration. The workflow feature of MolabIS enables users to insert or import a large number of data records via dynamic web forms. A form can consist of a sequence of sub-forms.

Data of very different formats (text, numerical data, images and archives as binary data) from the primary, final and descriptive data is stored in the central database, resulting in a transparent data handling independent of the data types. Instead of keeping files uploaded from the web browsers in the file system located in the web server all files are stored in a relational database as BLOBs. This approach avoids broken file links and storing many backup copies of data files. MolabIS allows lab users to store complete data sets of their projects: it captures both raw data and final data, as well as details of data operations and stores everything in a central database in a compact, coherent and uniform way. Figure 5 shows the data flows of the two methods supported in MolabIS for collecting data efficiently.

#### Workflow

**Figure 6:**
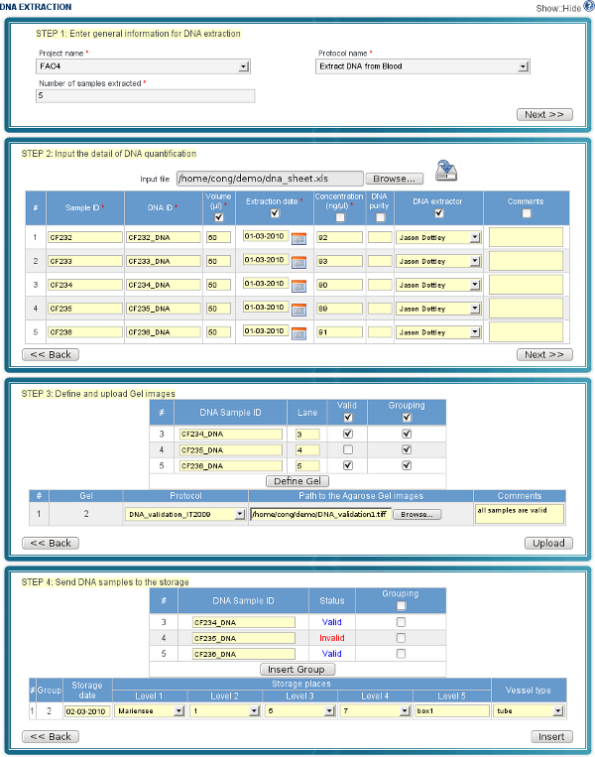
**GUI for uploading DNA samples**. The screenshot presents a sequence of web forms for inserting DNA samples into the database. Step 1: identify the number of samples which will be uploaded (e.g. 5); Step 2: enter data manually for all samples or upload data from the spreadsheet; Step 3: provide information on DNA validation and upload gel images (this step can be skipped if DNA samples are not checked); Step 4: select the storage locations of DNA samples.

**Figure 7:**
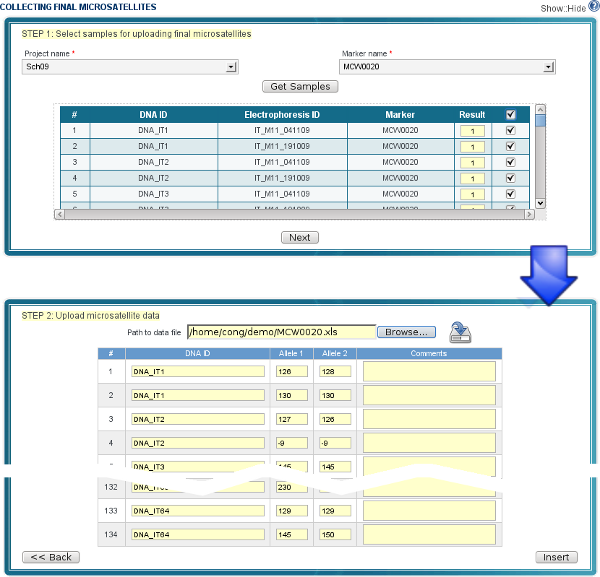
**GUI for importing microsatellite data**. The screenshot shows an example of improrting microsatellite data for 134 samples of a project. Step 1: identify samples of a project and select markers; Step 2: import microsatellite data from a spreadsheet and upload to the server.

Supporting the lab workflow is an important feature, as it allows users to easily keep track of their lab work and update their data in the database. Under the workflow (the left side of Figure 5), a scientist starts a project and then, step by step, interacts with the system to update the data until the project is finished. It is worth noting that data can be pipelined from one step to the next in the workflow. For example, samples exported in the step "Prepare Samples" in a spreadsheet format can be imported in the following step "Extract DNA" of the workflow. At each step, users are provided a web form or a sequence of sub-forms, for data entry. Forms are optimized for filling in data quickly (see Figure 6 and 7 for examples of two steps in the workflow).

#### Batch loading of historical data

In order to support data migration, "MLoader", an automation tool for bulk loading of historical data from previous projects has been developed. MLoader is a command line script written in Perl. It can be invoked at the back-end to import large datasets into a MolabIS database. All historical data must be available in electronic form to be accessed by the script (see the bottom right in Figure 5). In order to execute the script, a user must supply parameters and data spreadsheets. All parameters are indicated in a configuration file which is made up of file records (each record is a name/value pair). It means that the user needs to declare what kind of data should be imported into the database. MLoader provides different options for loading part of or all data of a project (e.g. loading only information on individuals, importing samples and final data, importing samples with both raw and final data, importing only final data). To prepare data spreadsheets, a user may fill in empty templates, which are predefined in a given format. The spreadsheets can be supplied in XLS, CSV, or ODS format.

### Data management

MolabIS not only keeps track of the workflow to capture and store different data types but also provides structured data handling capability i.e. it allows users to search for data across all projects, get back both raw and final data and modify any type of data stored in the database.

#### Search

Search functions are applied in the same manner for all web forms found under "Manage Data" and "Administration" in the main interface (Figure 4). A criteria based search mechanism is used, which allows the user to specify the criteria to be used in the search. Therefore, the search results can be extended or narrowed easily. Search results can be sorted according to any given field.

#### Data retrieval

**Figure 8:**
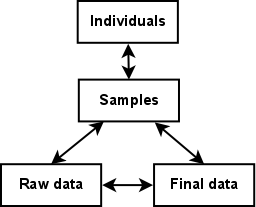
**Data association**. Relationship between associated data objects for keeping track of samples, and results.

In MolabIS, data objects are stored in a coherent manner, making data tracking much easier. The samples are considered the central entry point in the tracking model. Through the relationships among associated data objects as depicted in Figure 8 the system can locate related data. For instance, using the sample ID the user can retrieve information such as details on the sample itself, information on the individual from which the sample was collected, the storage location of the sample, details on the DNA extracted from the sample, raw data relevant to the sample, and final sequences obtained.

#### Data modification

MolabIS allows unrestricted data modification; lab managers can change any data field for codes, contacts, protocols, markers, storage places of samples in the lab. Scientists can update or delete all data objects stored in the database including individuals, samples, DNA, PCR amplifications, electrophoresis, sequence and microsatellite data of a project.

### Generating reports

MolabIS creates ready-to-print reports in PDF format based on user specified parameters. With a few mouse clicks, users can download PDF files to their computers. Thirteen predefined types of reports have been developed in MolabIS (see the list under the menu "Reports" in Figure 4). The system can provide lists of projects, contacts and individuals. It can make reports about information on samples or DNA, along with storage locations for a given project. Besides, statistical reports for sequences and microsatellites can be done for a particular marker, a certain project, or the whole lab. MolabIS also allows users to generate a report to sum up the data volume in the entire lab or make a chart of sample distribution of a project. Since the reports are based on templates, developers can easily modify the predefined types of reports.

### Exporting data

A further important feature of MolabIS is the export and conversion of final data to various formats required as input files for subsequent analyses, which is particularly useful for molecular labs working in the analysis of biodiversity.

#### Converting sequence data

**Figure 9:**
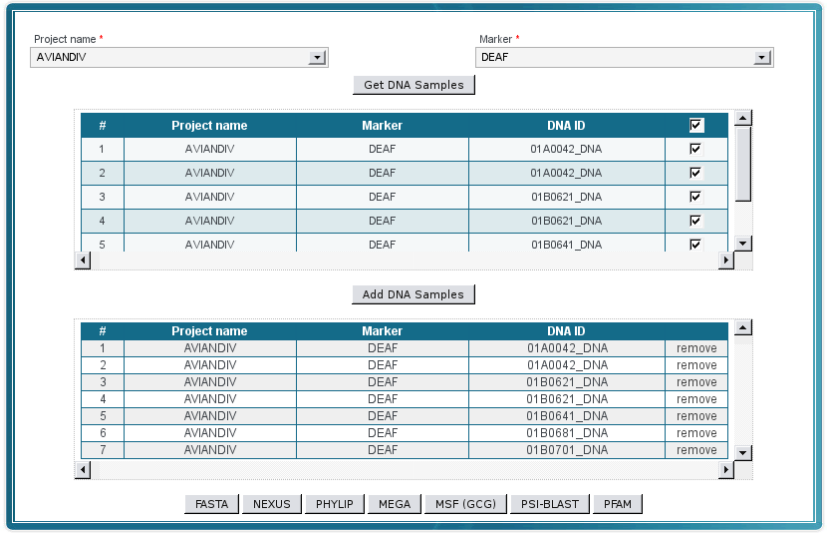
**GUI for extracting sequence data to various formats**. Sequences can be extracted and converted to different data formats. The GUI shows that users can select sequences from different projects for a specific marker to be merged into a multiple sequence file by clicking on a respective button at the bottom of the form. After pressing the button, the system will generate a file in a desired format which is available for download.

Different analysis tools expect different data formats. This requires scientists to convert sequences from a given format to another. MolabIS offers a tool to extract sequences stored in the database and to automatically convert them to various formats. The current version of MolabIS supports conversion of sequences to seven data formats: FASTA, NEXUS, PHYLIP, MEGA, MSF, PSI-BLAS and PFAM (Figure 9). Furthermore, the system can export sequences collected from different projects into a single file, which is available for download as required for instance in phylogenetic analyses. As an additional service, users can have their uploaded sequences in FASTA format converted to other formats by MolabIS.

#### Converting microsatellite data

**Figure 10:**
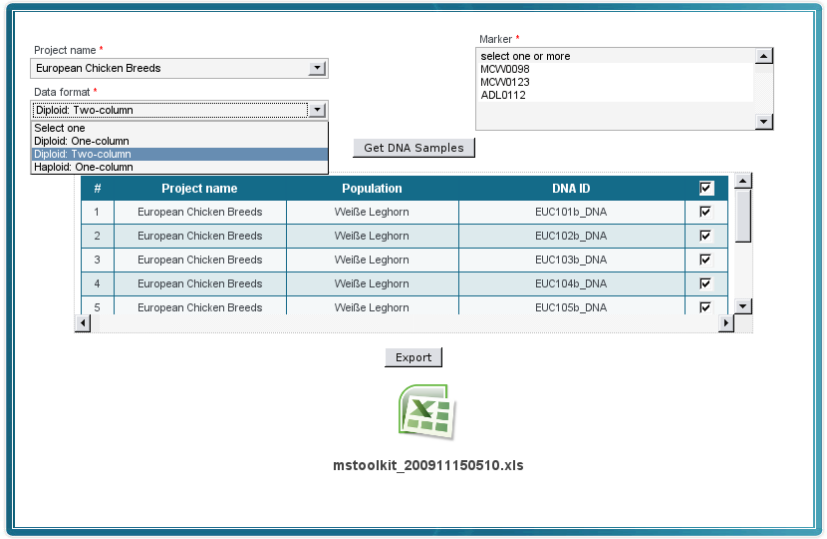
**GUI for extracting microsatellite data to various formats**. MolabIS can convert microsatellite data of a given project to various formats. The GUI shows that users can select a data format and a file format from drop-down lists for the output, identify markers and samples and click on the "Export" button to download the file.

Microsatellite data is frequently stored as a matrix in which rows represent samples and columns markers. Many of bioinformatics tools such as Microsatellite Toolkit [41] (an add-in utility for Microsoft Excel) require diploid or haploid microsatellite data as input files. Preparation of these input files may be tedious. Here, MolabIS helps by extracting microsatellite data of samples from different projects and by exporting all to a single file. It allows the user to select one of three types of data formats (one-column diploid, two-column diploid, one-column haploid) for exporting. In addition, the user can choose Excel or CSV (Comma Separated Values) as the file format of the output. This process is depicted in Figure 10.

### Performance and scalability

By using Postgres, MolabIS obviously meets the requirements regarding time and space complexity mentioned in [21]. It can store large amounts of data and is only limited by the hardware configuration of the server. The software has been tested to ensure that it can be used by multiple users at the same time in a LAN, as well as the Internet. MolabIS runs without performance issues even when used by 10 simultaneous users.

To evaluate the performance and scalability of MolabIS, we have done three tests on three databases but with different sizes (1,000, 10,000, and 100,000 records of samples, respectively). The tests were conducted on a computer with an Intel(R) Core(TM) i5 2 × 2.30 GHz processor and 6 GB of RAM, running Kubuntu 11.04 and using Postgres 9.0. All tests used the same test cases, which are typical queries in a production mode. For each test, a test case was executed and benchmarked ten times at the front-end to calculate the mean response time. The results are reported in Table 4 showing response times in the order of seconds, thereby allowing the users to rapidly interact with the system. As expected, the response times are independent of the size of the database indicating that MolabIS scales well. Indeed, the differences in the response time among tests are insignificant (less than 0.30 seconds for each test case).

**Table 4:** Performance results of MolabIS

Test case	Number of samples in database		
	
	1,000	10,000	100,000
Insert 50 samples into database	6.55 ± 0.32	6.69 ± 0.27	6.47 ± 0.34
Retrieve 500 samples from database	1.62 ± 0.06	1.67 ± 0.06	1.91 ± 0.05
Export 7,000 microsatellites to CSV	2.16 ± 0.11	2.11 ± 0.10	2.10 ± 0.10

The response time is measured in seconds at the front-end of a client machine in a LAN. For each test case, the mean response time and standard deviation is calculated from the response times of ten runs. The databases consist of approximately 1,000, 10,000 and 100,000 samples for the three tests, respectively.

## Discussion

MolabIS was developed to overcome the challenges of molecular genetics labs in the context of data management as defined in the requirement section. In the following, we summarize how MolabIS addresses the requirements listed in the section "Background".

**R1: **While other information systems are often designed to collect data of either DNA sequencing projects or microsatellites genotyping projects, MolabIS is the only system to support both.

**R2: **MolabIS can manage information on individuals in plants and animals from any species and breed.

This feature is not supported in other information systems.

**R3: **The functionality of sample management in MolabIS is considered a complete software package for the storage and management of samples. MolabIS allows to track a large number of samples of different types. It provides a five-level hierarchical storage scheme ensuring the flexibility in the representation of physical storage locations of samples and DNA in different labs. The lab manager can define a new location, update and delete existing ones at any storage level.

**R4: **The workflow, one important feature in MolabIS, supports the experimental workflow in the wet lab efficiently and organizes the data entry accordingly. Data is pipelined from one step to the next in the workflow. At each step in the workflow, the details of lab work such as PCR amplification, PCR validation, and electrophoresis are recorded. This feature also highlights the difference between MolabIS and other systems, which only support importing final data.

**R5: **All data operations can be performed via a standard web browser including Internet Explorer 7+, Firefox 3.0+ and Safari 3+ running under a variety of operating systems. The Ajax technology used in MolabIS allows to create an interactive user interface, which has the quality of desktop applications. The users can search, view, update, and delete their data in a single form without switching screens. Raw data (e.g. gel pictures, chromatogram data) is stored independent of architectures of the sequencers. Therefore, MolabIS can manage all electrophoresis products, which can be obtained from different sequencers, in a uniform way. The import functionality of MolabIS has considerably enhanced the process of data entry. The details of samples and DNA can be imported in various file formats, such as .xls, .ods, or .csv. Moreover, sequence and microsatellite data can be imported into the database. Additionally, every data entry form can store additional information in a comment block thereby allowing MolabIS to function as a filing cabinet.

**R6: **JasperReports, an embeddable open source Java reporting library, is integrated in MolabIS to provide an effective reporting solution. The report templates are compiled with parameters specified by the user to extract data from the current database and generate the report. Although the system currently supports generating reports in PDF format, the report templates can easily be extended to other formats.

**R7: **MolabIS supports the retrieval of final data, as well as original files of raw data of any project. In addition, final sequences and microsatellites can be converted to various formats.

**R8: **Developed as a web application, MolabIS can be installed and used in a LAN or Internet, thus allowing many users to access the system simultaneously. Under the access rights control of MolabIS, data is used and shared in a secure manner. MolabIS is well-suited for localization. The text, labels, and context help in all web forms are read from an ASCII file (text file) which can be edited by any text editor.

**R9: **We used virtualization technology to package and deploy the application. Hence, the MolabIS appliance can be installed on different platforms (e.g. Linux, Windows). The installation process itself amounts to downloading the appliance file, installing the virtual player and running the appliance under the virtual player without any knowledge about its operating system or other software components. Under the GNU General Public License, MolabIS can be downloaded, installed and used free of charge. This contrasts the traditional installation which starts with the installation and configuration of DBMS, web server, application framework and software components, thus requiring IT experts, who usually are not present in most labs.

**R10: **Loading data from previous projects can be carried out in a batch loading mode. The MLoader can be used to load large amounts of data collected and stored over the years. It executes a sophisticated system of foreign key loading and rollbacks. This facilitates the detection of similarly spelt keys and the restoration of origin data for wrong data loading.

The above list indicates that the requirements, as stated in the first section of this paper, have been met. Our software package was tested by third parties who are independent of the development of the application. Thorough testing has been carried out, in order to check for both technical bugs and missing functionality. Moreover, a user guide is available and released along with the software.

## Conclusions

The development of MolabIS has solved the problems described in the first part of this paper. MolabIS is a web-based integrated information system which can be used to store, manage and handle data of DNA sequencing and microsatellite genotyping workflows. All operations can be done via a standard web browser running on any operating system. Developed as an open source software package, MolabIS takes advantage of other open source components. It brings benefits to both researchers and lab managers. For researchers, their data is stored safely with high reliability. In collaborative projects, the data can be shared in a secure manner. The system helps to reduce the workload and the time needed for searching and preparing data for subsequent lab work steps. The conversion of data formats is performed easily, thus saving time and avoiding human errors. For lab managers, MolabIS ensures long-term data storage and monitors the progress of different projects carried out by various lab members. In fact, MolabIS supports full documentation of genotyping and sequencing experiments, even with short term lab users (e.g. students or visiting scientists) and different genotyping platforms. With its general data model, MolabIS meets common requirements of various molecular genetics labs working in biodiversity. Released under the GNU General Public License, MolabIS can be downloaded, modified and used freely. MolabIS is distributed as an appliance in which all components and services are installed and pre-configured. Being a ready-to-use appliance, it can be run on different platforms by using a free player such as VMWare Player or VirtualBox with minimal installation effort.

Rapid advances in molecular genetic technology have led to a quick adaption of high throughput genotyping for SNP and NextGen Sequencing. Future releases of MolabIS will have to address this development, possibly also adding support for other molecular markers like AFLPs, which are still being used in many small labs, especially in developing countries. To accommodate these changes, the data model will have to be expanded, while preserving the core part of the sample management and all current functionality.

## Availability and requirements

The source code, user guide and appliance of MolabIS are freely available at the project homepage http://www.molabis.org. We also provide a live demo for users who want to evaluate MolabIS without installation. Release notes and other information will be also updated on the project homepage.

**Project name: **MolabIS

**Project homepage: **http://www.molabis.org

**Operating system: **Platform independent

**Programming language: **Perl **Database: **Postgres

**License: **GNU GPL

## Authors' contributions

CT designed the data model, implemented the software, and wrote the manuscript. LG evaluated and enhanced the usability of the software and wrote the user's guide. EG initiated and supervised the project. BM co-supervised the project and revised the manuscript. All authors edited, read and approved the final manuscript.

## Supplementary Material

Additional file 1 **Source code of MolabIS**. The source code of MolabIS is provided as a Zip file. Click here for file
