# Supplementary material for: MolabIS - An integrated information system for storing and managing molecular genetics data
Source: BMC Bioinformatics. 2011 Oct 31;12:425. doi: 10.1186/1471-2105-12-425 (PMC3268772; doi:10.1186/1471-2105-12-425)
Supplement: Additional file 1 — Source code of MolabIS. The source code of MolabIS is provided as a Zip file. [file 1471-2105-12-425-S1.ZIP › molab/templates/samples.html]

|  |  |  |  |  |
| --- | --- | --- | --- | --- |
|  |  |  |  |  |
| > | > |  |  |  |

  


|  |  |  |  |
| --- | --- | --- | --- |
|  |  |  |  |
|  | 0 |  |  |
|  | | | |

  


|  |  |
| --- | --- |
|  | :: |

|  |  |  |  |  |
| --- | --- | --- | --- | --- |
|  |  |  |  |  |
|  | \*      > | \*      > |  |  |
|  | \* | \*      > |  |  |
|  | \* | \*      > |  |  |
|  | \* | \*      > |  |  |
|  | \*      \*     > | <TMPL\_VAR NAME=comment> |  |  |
|  | \*   \|  \|  \|  \|  \|  \| \| --- \| --- \| --- \| --- \| --- \| \|  \|  \|  \|  \|  \| \| > \|  \|  \|  \|  \| | |  |  |
|  |  |  |  |  |
|  | /> | | | |
|  |  |  |  |  |
