# Supplementary material for: MolabIS - An integrated information system for storing and managing molecular genetics data
Source: BMC Bioinformatics. 2011 Oct 31;12:425. doi: 10.1186/1471-2105-12-425 (PMC3268772; doi:10.1186/1471-2105-12-425)
Supplement: Additional file 1 — Source code of MolabIS. The source code of MolabIS is provided as a Zip file. [file 1471-2105-12-425-S1.ZIP › molab/templates/main.html]

MolabIS: A Labs Backbone for Storing, Managing and Evaluating Molecular Genetics Data

 >

:

MolabIS Beta, Under GPL, by Truong Van Chi Cong - All Rights Reserved
