# Supplementary material for: MolabIS - An integrated information system for storing and managing molecular genetics data
Source: BMC Bioinformatics. 2011 Oct 31;12:425. doi: 10.1186/1471-2105-12-425 (PMC3268772; doi:10.1186/1471-2105-12-425)
Supplement: Additional file 1 — Source code of MolabIS. The source code of MolabIS is provided as a Zip file. [file 1471-2105-12-425-S1.ZIP › molab/www/index.html]

xml version="1.0" encoding="utf-8"?


MolabIS: A Labs Backbone for Collecting, Managing and Evaluating Molecular Genetics Data


<body>
<h1>MOLABIS</h1>
<p> A Lab Backbone for Collecting, Managing and Evaluating Molecular Genetics Data<br />
Click to proceed to the
<a href="cgi-bin/login.cgi" title="MolabIS Home ">MolabIS Home</a>
</p>
</body>
